# Natural Ingredients-Based Gummy Bear Composition Designed According to Texture Analysis and Sensory Evaluation In Vivo

**DOI:** 10.3390/molecules24071442

**Published:** 2019-04-11

**Authors:** Ugnė Čižauskaitė, Greta Jakubaitytė, Virgilijus Žitkevičius, Giedrė Kasparavičienė

**Affiliations:** 1 Institute of Pharmaceutical Technology, Lithuanian University of Health Sciences, A.Mickevičiaus g. 9, LT-44307 Kaunas, Lithuania; giedre.kasparaviciene@lsmuni.lt; 2 Department of Drug Technology and Social Pharmacy, Lithuanian University of Health Sciences, A.Mickevičiaus g. 9, LT-44307 Kaunas, Lithuania; greta.jakubaityte@gmail.com; 3 Department of Drug Chemistry, Lithuanian University of Health Sciences, A.Mickevičiaus g. 9, LT-44307 Kaunas, Lithuania; virgilijus.zitkevicius@lsmuni.lt

**Keywords:** gelatin, chewable dosage form, gummy bear, experimental design

## Abstract

The increased interest in functional materials of natural origin has resulted in a higher market demand for preservative-free, “clean label”, or natural ingredients-based products. The gummy bear food supplements are more acceptable to consumers and have fewer limitations compared to other dosage forms. The aim of our study was to produce natural ingredients-based gummy bear composition, and evaluate the influence of the selected ingredients on the product’s textural properties, its acceptance in vivo, and the gummy bear’s quality. The optimal base composition was determined using a surface response design: gelatin 4.3 g and agave syrup 6.3 g. The investigated sweeteners did not affect the textural properties (*p* > 0.05). However, further studies demonstrated that a 100% increase of agave results in up to 27% higher flexibility (*p* < 0.05). The addition of calcium and cholecalciferol reduced firmness by 59.59 ± 1.45% (*p* < 0.05). On the other hand, acai berry extract had no significant effect. The presence of calcium resulted in a decreased smell and taste; however, the data indicated that experimental texture analysis is a more accurate technique than in vivo evaluation. The acai berry extract did not improve all of the tested sensory properties. We can conclude that the suggested gummy bear base can be supplemented with various active ingredients and commercialized, though further studies are needed to investigate the other natural sources to mask the unpleasant taste of active ingredients and avoid water loss.

## 1. Introduction

In the 21st century, the interest in natural ingredients-based food supplements, pharmaceuticals, and functional ingredients has amplified [1]. According to the studies of Zhu & Woerdenbag and Choochote et al., the application of phytochemicals derived from herbs and fruits is a potential alternative to synthetic active ingredients to enhance food quality, minimize toxicity, and ensure environmental safety due to the nontoxic biodegradation [2,3]. It is known that the oral route is the most convenient route for the administration of active ingredients in food supplements, functional food products etc., due to the highest component of compliance mainly being the pediatric and geriatric patients. It is regarded as the most economical and safest method of drug delivery [4]. However, the development of dosage forms and taste-masking of bitter, salty, or sour bio-actives administered orally, especially if the product has to be suitable for children, are formidable challenges for formulation scientists. According to EMA recommendations, liquid formulations are the most appropriate for patients younger than eight years old [5]. However, the dosage of bio-actives in liquid products is limited due to their solubility and a lot of additives, such as sweeteners, buffers, preservatives etc., must be used to ensure physical, chemical, and microbiological stability and improve the flavor [6]. A more sophisticated formulation approach is gummy bears based on natural materials with incorporated active ingredients. The gummy bears, as is the case for all other chewable food products, are only available for children and adults without a chewing disorder and dysphagia, but can still broaden the consumer market due to the wide list of benefits.

It has been reported that a gummy bear’s base usually consists of the jellifying agent (pectins, modified starch, gelatin etc.) and sugars, where water-soluble ingredients can be dissolved and the insoluble ones are suspended in the viscous matrix [7,8]. Therefore, the application range of gummies in the pharmaceutical and food industry as a novel drug delivery system, which is more acceptable to children and some adults due to the confectionary appearance and taste, is wide. Some studies have determined that the composition of gummy bears, especially the concentration and origin of gelling agent and sugars, has a significant impact on the rheological properties of the product [8,9,10]. An increasing amount of gelatin in a food matrix has been shown to increase the thickness of the product associated with a reduction in the perception of flavor [8]. According to L. DeMars and R.G. Ziegler, gelled products are easily made on a gelatin base though opportunities still exist for improving and modifying their texture since various possible textural changes have never been adequately defined and quality evaluation assays of gummy bears have been suggested and performed [11].

However, the use of gummy bears as food supplements or nutraceuticals is limited due to the partially or fully hydrogenated oils present in the composition and high sugar contents, usually in excess of 50% plus sugar, which results in little nutritional value and contributes to health problems such as an increased risk of developing cardiovascular and heart disease [12]. The benefits and harm ratio of gummies with sugar syrup is questionable considering diabetic patients and children with attention-deficit/hyperactivity disorder [13,14]. Since sweeteners also play a main role in affecting not only the sensory properties of the product, but also the viscosity of the aqueous phase and the water activity, their choice must be considered whilst paying attention to the health benefits as well [8]. The data correspond with the results of another study, which has determined that sugar alternatives (xylitol, mannitol) in gummy bears are successfully used as sweeteners and even prevent dental carries [15]. Therefore, it is relevant to produce a superior gummy bear base from the health perspective composed of natural ingredients, which could be further used to incorporate various active ingredients and additives.

The selected active ingredients to incorporate into the gummy bear base were calcium carbonate and vitamin D_3_ due to the high ratio of population in need, particularly elderly women and children [16]. However, the challenge with calcium-containing products is “chalky”, bitter-salty taste masking [17,18]. It has been suggested that the unpleasant taste of mineral salts could be hidden using a high percentage of sugars and their substitutes because healthier alternatives such as the addition of natural flavors and fruit juice have not been very successful according to consumers’ evaluation [19,20]. Our approach to solve the taste and color problem is the use of acai berry extract, which is rich in anthocyanins and anthocyanidins and possesses various beneficial effects on human health, such as antioxidant and anti-inflammatory activity; reduces the risk of cardiovascular disease; and has a hypocholesterolemic effect [21,22]. According to Constant et al., a color additive prepared from acai was more efficient in comparison with other plant-derived substances [23]. In addition, it has a pleasing flavor reminiscent of boysenberry or cherry with chocolate overtones [24]. The positive effects of the acai berry were stated in various studies in vitro, where its use as a nutraceutical was suggested; however, there is a lack of evidence on its possible applications in the food and pharmaceutical industry as a natural colorant or flavoring agent [22].

Therefore, the aim of our study was to produce natural ingredients-based gummy bear composition, evaluate the influence of the selected ingredients on the textural properties of the product and its acceptance by the consumers, and propose analytical assays for the evaluation of the gummy bear’s quality. The optimal gummy bear base composition with ingredients chosen considering their effect on human health could be further used in confectionery, food supplements, or pharmaceutical manufacturing. 

## 2. Results

To evaluate the influence of various commercially used sweeteners for gummy bear manufacturing, a pilot study was carried out in order to choose the best technological and functional option for the gummy bears containing a pork gelatin base. The chosen sweeteners were sugar and its substitutes due to the considerable application for diabetic patients and children with attention-deficit/hyperactivity disorder [13,14]. The results demonstrated that no significant changes in various textural properties were observed between the sweeteners, so all of the tested materials could be used in gummy bear manufacturing (Table 1). The obtained data were in contradiction with the theory provided by other researchers; that sweeteners play an important role in affecting sensory and rheological properties of the product [8]. It was determined that the viscosity of various sweeteners (corn fructose, agave, and sugar syrups) is a particular divergent property (sugar syrup > agave syrup > corn syrup), which leads to the conclusion that the choice of these sweeteners in the confectionary or dough manufacture impacts the viscosity and other textural properties of the products [25]. Strode and Galoburda observed that the substitution of sugar with agave syrup in chocolate manufacturing had a positive influence on the quality of the product: a smaller size of sugar crystals, lower stress yield, and increased viscosity [26]. Since our results were inconclusive, the choice of sweetener was made according to the data found in the literature: the characteristics of the product. Potential harm to the users was taken into consideration as well. The reduction of sugar is still a challenge for the food and pharmaceutical industry due to the importance of its provided functional, sweetening, and texturizing properties, but it can be achieved using sugar alternatives or high-intensity or bulk sweeteners [27]. Xylitol is a well-known sugar substitute and many studies have proven its beneficial effect in terms of glucose tolerance, serum insulin concentration, obesity development prevention, protection of renal and hepatic functions etc., though higher intakes and higher dosages of the sugar alcohols cause several gastrointestinal discomforts, including diarrhea [28,29,30,31]. Recently, agave syrup has gained popularity as an alternative to traditional sweeteners due to its comparably low glycemic index (11 ± 1) and its status as vegan [32,33]. Agave syrup contains a high carbohydrate content (>95%), with the major component being fructose, which has been reported to range from 55.6% to 90% [32]. It has been stated that some of the constituents of agave can be used as part of the dietary strategy to ameliorate the metabolic abnormalities observed in obese subjects and in comparison to sucrose, agave may have a positive influence on glucose control [34,35]. Since no information regarding the toxic effect of agave syrup has been found, it was selected as a sweetener for gummy bear manufacturing and used throughout the experiment. 

Further on, 13 experiments according to the mixture design were carried out to determine the influence of the components used to form a gummy bear base on the selected responses: firmness, strength, and hardness. Significant differences were found between the mixtures. To obtain more detailed information, predicted equations are shown in Table 2.

**Figure 1:**
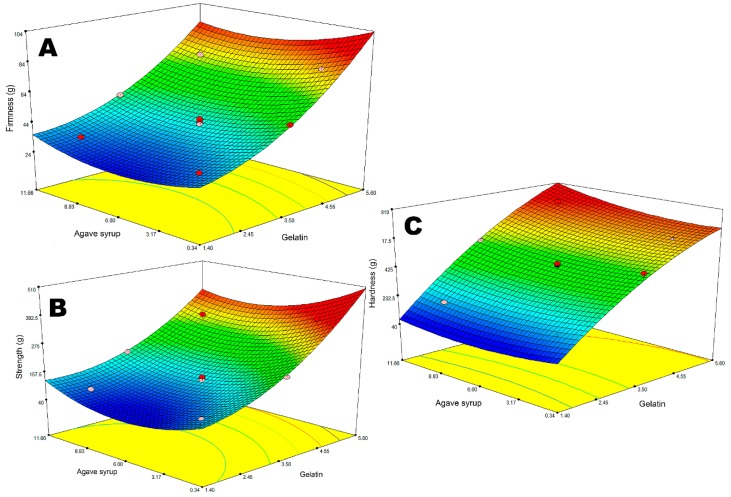
The influence of variables (agave syrup and gelatin) on the textural properties (firmness (**A**), strength (**B**) and hardness (**C**)) of gummy bears.

The results demonstrated that both of the ingredients used to form the gummies’ base had a significant influence on the firmness and strength of the product, though only gelatin affected the hardness of the chewable dosage form. It was observed that double the amount of agave syrup in the composition with the same concentration of gelatin results in a 7–14% lower firmness and 17–27% increase in flexibility (*p* < 0.05). There were no significant changes observed in the hardness of the gummy bears. In contrast, recent studies have determined that the addition of sweeteners (xylitol) at relatively low concentrations (3–5%) increased the firmness of the gelled structure. However, the further addition resulted in a decrease of viscosity, probably due to the gel network formation disturbance [36]. A direct correlation was observed between gelatin concentration and firmness, strength, and hardness. As expected, when the amount of structuring agent in the formulation changes from 2.0 to 3.5 and 5.0 g, the firmness of the product increases from 34.31 ± 1.12 to 49.76 ± 2.35 and 73.37 ± 3.08 g, respectively (Figure 1). The same trend was observed during the flexibility test. The strength of a sample containing 3.5 g of gelatin increased two-fold compared to 2.0 g (*p* < 0.05). Since hardness and firmness are similar textural properties and both can be related to the viscosity index, there was no surprise when decreasing the jellifying agent concentration by 3.0 g resulted in a 73.36 ± 3.51% lower hardness of a gummy bear. Some studies have evidenced that the concentration of gelling/thickener agents, as well as of sugars, can affect the textural properties of gelled products, like candies, gummy bears etc., so the obtained data correspond with the results achieved by other researchers [8,9,10].

Numerical optimization within the software was used to determine the optimum mixture conditions. It was carried out using the desirability (multiple response) method. This approach includes targeted values, maximized or minimized values of the outcome, and the preferences of their importance in order to determine the relationship between the selected variables and the predicted and desired responses [37]. The aim of this study was to find the most suitable composition of the gummy bear’s base with good textural properties, so firmness and hardness were set as responses of major importance, and flexibility was set as of medium importance. Numerical optimization modulated a solution according to the obtained results in the experimental design matrix and desired conditions with a desirability value of 0.914. The results of the empirically predicted gummy bear base evaluation showed that the experimental values were in agreement with the predicted values (*p* > 0.05) (Table 3).

In the second stage of the experiment, four different gummy bear compositions were made using the optimized gummy bear base (Table 4). Grapefruit seed extract was chosen as a natural preservative due to its well-known antimicrobial and antifungal properties and potential use in the food and cosmetics industry [38,39]. Sorbitol was used as a plasticizer in order to achieve a barrier to avoid water loss and apple acid was needed to modify the pH [40]. The concentration of additives (sorbitol, apple acid, and grapefruit seed extract) was kept as a constant throughout the experiment. Calcium carbonate accompanied by cholecalciferol solution were selected as active ingredients of the gummy bears due to the large demand in the market, particularly by post-menopausal women and children [16]. It is generally accepted that adequate calcium and vitamin D_3_ intake, for which a deficiency reduces calcium absorption, is an important contributor to modestly lowering the risk for bone fractures and attaining peak bone mass in adolescence [41,42]. Calcium salts are described as salty, bitter, sour etc., so the chewable dosage forms containing calcium salts usually have an unpleasant taste [18]. The taste issue is solved by adding several sweeteners, natural flavors, colorants etc., though according to Rees and Howe, the flavoring additives do not always improve the acceptability of the preparation [19]. In the current study, acai berry extract, rich in antioxidants, was used to dye the gummy bears and improve their taste.

**Figure 2:**
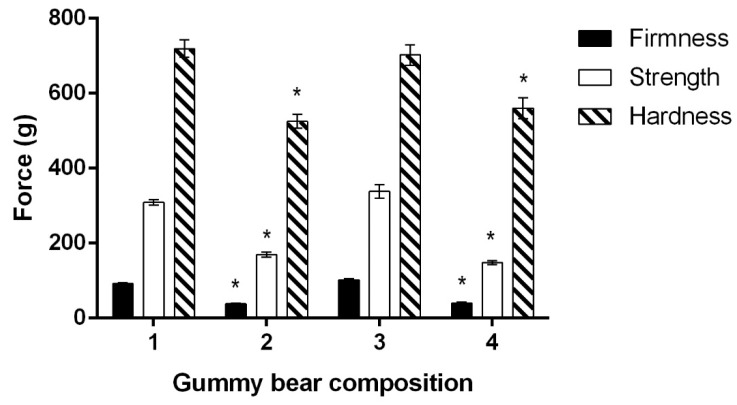
The influence of gummy bear composition on the textural properties. * *p* < 0.05 vs. 1, 3 compositions.

Before the organoleptic properties evaluation of the four gummy bear’s compositions, the physicomechanical properties were determined (Figure 2). There were no statistical differences in firmness, strength, and hardness observed between composition No. 1 and No. 3, which was the same for No. 2 and No. 4. Therefore, we can conclude that the acai berry extract did not affect the textural properties of the gummy bear composition; however, other researchers have suggested that the influence of acai berry on the textural properties and even moisture loss during storage may depend on acai berry processing [43]. Censi et al. determined that the addition of an acai berry extract did not influence the density of the composition containing a thickening agent, so our results correspond with the data obtained in this study [44].

The addition of calcium carbonate and the aqueous solution of cholecalciferol reduced the tested textural parameters: firmness by 59.59 ± 1.45%, strength by 50.67 ± 5.53%, and hardness by 23.59 ± 3.37% (*p* < 0.05). Presumably, the hardness of a gummy bear was affected the least due to the physicochemical properties of calcium carbonate. It has a very low water solubility (6.8–15 mg/L), so it was suspended in the gelatin-based viscous liquid while making the gummy bears [45]. However, the suspended particles of calcium remained solid so they could interfere with the penetration force measurement during the hardness determination. The results of a study conducted by Valencia et al. are in contradiction with our data: the amount of calcium did not affect the hardness of soft candies, though it was noted that other textural properties, such as springiness and chewiness, were twice as big compared to the control [17].

**Figure 3:**
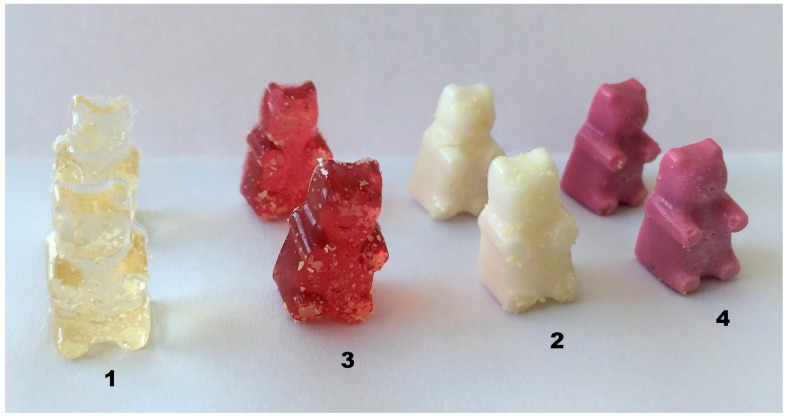
The view of the four gummy bear compositions. See the explanation of 1, 2, 3, 4 in Table 4.

**Figure 4:**
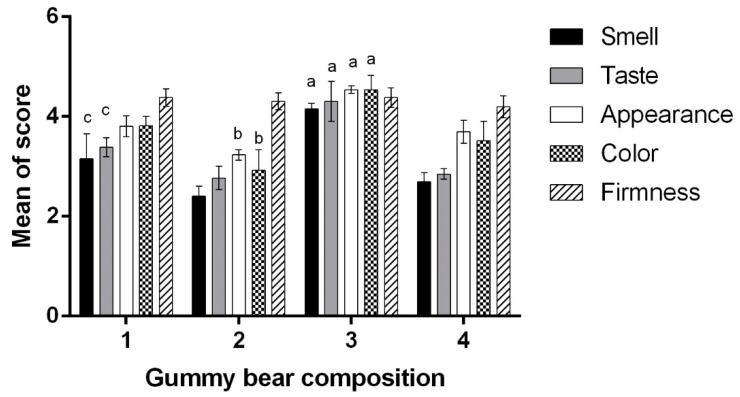
The influence of gummy bear composition on the textural properties. ^a^ *p* < 0.05 vs. 1, 2, 4 compositions; ^b^ *p* < 0.05 vs. 1, 4 compositions; ^c^ *p* < 0.05 vs. 2, 4 compositions.

In the next stage of the experiment, human volunteers evaluated the organoleptic properties of the four different gummy bear compositions (Figure 3). The results are presented in Figure 4. The firmness evaluation in vivo did not comply with the texture analysis results: the participants did not notice any difference in firmness between the gummy bear samples. Similar results were achieved by Periche et al., so it was concluded that analytical texture analysis methods are more accurate, especially when the difference in hardness is relatively small [46]. The highest color evaluation achieved formulation No. 3 (4.54 ± 0.28) containing acai berry extract. According to Khoo et al., acai berries, rich in anthocyanins, which are red colored pigments soluble in water, should be used in the food industry due to their positive health effects as a natural colorant [21]. However, the addition of calcium carbonate significantly lowered the color score by 22.47 ± 0.09% and finally, there was a no color difference between the calcium-containing gummy samples with or without the acai berry extract. Our results are in contradiction with the findings of Valencia et al., where the addition of calcium did not result in any significant color changes [17]. Later on, a direct correlation (0.9915) between appearance and color indications was observed, though the addition of acai berry extracts to the gummy bears with calcium carbonate improved the appearance score by 14.24 ± 4.2% (*p* < 0.05). The data correspond with the opinion of other researchers, who declared that the color additive prepared from acai was more efficient in comparison with the color additive from grapes and other plant substances due to the longer stability of the dye and better product appearance [22,23]. 

Divalent salts are known to have an unpleasant complex taste, which is difficult to hide [18]. According to the obtained results, the addition of calcium carbonate to the composition of gummy bears negatively affected the taste of the product: it decreased by 25.69 ± 1.8% compared to the control (No. 1) and by 33.72 ± 3.8% compared to composition No. 2 (*p* < 0.05). Some researchers determined that the right approach to a calcium-containing composition is to affect sweetness enhancement, remove bitter and metallic tastes and aftertastes, and minimize the “chalky” flavor; however, the suggested solutions are mainly a high percentage of sweeteners [19,20]. The highest smell value gained the composition No 3 (4.15 ± 0.12), which contains acai berry extract (*p* < 0.05). This may occur due to the taste of acai berry, whose extract or pulp is widely used in the Amazon region to flavor food [22,47]. On the other hand, the addition of calcium carbonate decreased the smell value by 35.18 ± 4.20% (*p* < 0.05). The mean smell score of sample No. 1 was relatively higher than those of No. 2 and 4; however, it was not statistically significant. The addition of aqueous vitamin D_3_ solution possibly had an impact on the decrease in smell value: it possesses a bitter smell [48].

In assessing the quality of the gummy bears, we suggest following the requirements for chewable dosage forms (tablets) according to the European Pharmacopoeia since there are no guidelines for gummy evaluation yet. For the quality evaluation, composition No. 4 containing calcium carbonate, aqueous vitamin D_3_ solution, and acai berry extract was selected. It was found that at t_0_, the concentration of active ingredient and mass uniformity of the gummy bears did not exceed the allowed deviation (5%) (Table 5). The dissolution time deviation is not applicable.

According to the obtained results, the mass of the gummy bears decreased over time. The moisture loss is one of the main issues while making gummy bears because the composition usually contains a high percentage of water. Edwards and Vercet have suggested that the recommended moisture content for the gummy bears should be around 24% [49]. Based on the data provided by other researchers, the formulations containing gelatin as the thickening agent have a higher initial moisture content and are more sensitive to water loss compared to pectins or gumi arabic [8]. As in our study, the weight of the investigated formulation decreased by 20.7 ± 1.3% after seven days of storage at room temperature and by 43.76 ± 2.1% after two weeks (*p* < 0.05). Other research has determined that the interactions of the sugar choice (fructose, isomaltose etc.) and the percentage of the gelatin used have a significant impact on the moisture content, as well as the water activity, which may result in microbial growth and a low stability of the product [46].

## 3. Materials and Methods

### 3.1. Materials

Gelatin (pork) was purchased from Fluka Analytical. Sorbitol, xylitol, sucrose, and carnauba wax were procured from Sigma-Aldrich (St. Louis, MO, USA). Agave syrup was purchased from “The groovy food company” (London, UK). Grapefruit seed extract was obtained from Akamuti Limited (Lindeilo, UK) and apple acid (>98%) was purchased from Alfa Aesan—A. Johnson Matthey Company (Karsruhe, Germany). The acai berry extract was given by Xian Tonking Biotech Co. (Xi’an, China). Calcium carbonate was procured from Car Roth GmbH (Karsruhe, Germany) and the aqueous vitamin D_3_ solution (Aquadetrim^®^) was purchased from Medana Pharma SA (Sieradz, Poland).

Hydrochloric acid (37%), ammonium hydroxide (>25%), ammonium chloride-ammonium hydroxide buffer solution, methyl red (>95%), eriochrome black, and ethylenediaminetetraacetic acid (EDTA) were purchased from Sigma-Aldrich (St. Louis, MO, USA). Distilled water was used throughout the experiment.

### 3.2. Gummy Bears Technology

Gummy bears were manufactured by molding a prepared mass in a silicone mold and cooling it down to room temperature. 

The bases of the gummies were prepared by the following steps. The pork gelatin was added into the distilled water and left to swell at room temperature (22 ± 2 °C) for 30 min. The amounts of base materials were added according to the surface response design matrix (Table 6). The water/gelatin mixture was heated in a water bath W16 (Harry Gestigkeit GmbH, Dusseldorf, Germany) at 50 °C until the homogeneous viscous liquid formed. Further on, the sweetener was added to the composition, followed by other additives, such as calcium carbonate, grapefruit extract, apple acid, aqueous vitamin D_3_ solution, acai berry extract, and sorbitol, if required. The prepared mass was well-mixed, filtered through a double cheesecloth, and poured into the silicone mold, which was powdered with 1 g of carnauba wax before the experiment. The mold was left at room temperature (22 ± 2 °C) to dry for 48 h [8].

### 3.3. Preparation of Sugar and Xylitol Syrups

The sugar and xylitol syrups were made by adding 60 g of sweetener to 40 g of distilled water and heating the mixture until it reached the boiling point (105–110 °C). After 5 min, the prepared hot viscous solution was filtered through a three-layer cheesecloth. The solution was weighted and adjusted to 100 g if necessary [50].

### 3.4. Experimental Design

The experimental design of gummy bears was conducted using Design Expert (version 7.0, Stat-Easy Inc., Minneapolis, MN, USA). A set of candidate points in the design space was selected using the D-optimal surface response design. Two independent variables, namely agave syrup and gelatin, were chosen. The water content was adjusted according to the gelatin concentration up to 40 g (Table 3). The preliminary experiments were carried out before the current study in order to determine the ranges of gelatin (A) and agave syrup (B) (Table 6). The selected responses were firmness, strength, and hardness. The statistical analysis tables were generated using analysis of variance and the significance of all the variables was determined by calculating the F value while the *p* criterion was ≤0.05. Numerical optimization according to the multiple response (desirability) function was carried out. The predicted optimal concentration of the mixture and the compliance with the predicted response were verified by conducting experiments at the determined concentrations of variables using the same experimental conditions. 

### 3.5. Texture Analysis

Texture analysis was performed using the TA.Xtplus Texture Analyzer (Stable Micro Systems, Godalming, UK) at room temperature (22 ± 2 °C).

**Figure 5:**
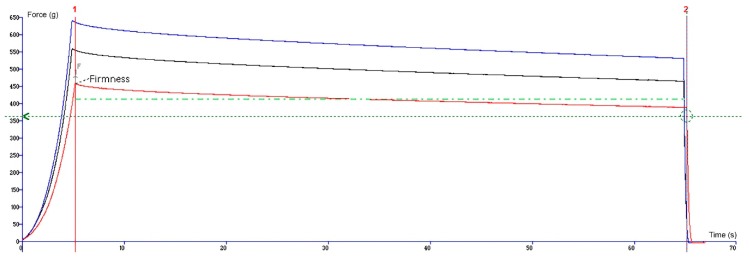
A graphical view of the compression test. The lines represent the three replicates of the sample.

For the compression test, an analytical probe p100 was forced down onto each gummy bear sample at a defined rate (1 mm/s) with a trigger force of 5.0 g using a load of 5 kg to measure the compression force (firmness). Once the trigger force was attained, the probe proceeded to compress the sample to 20% of its original height. It was held at this distance for 60 seconds and then withdrew from the sample to its starting position. The bellow plot illustrates a force-time (or distance) curve, which shows the characteristics of gum firmness and springiness (Figure 5). The experiment was performed three times, and the results are presented as the mean ± standard deviation. 

**Figure 6:**
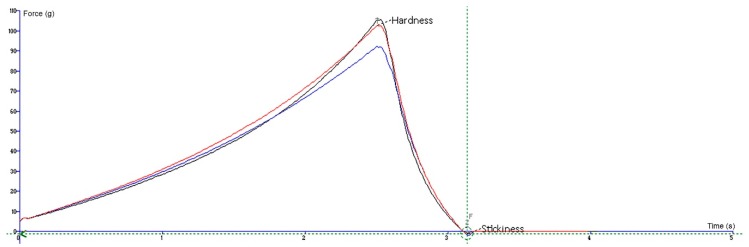
A graphical view of the penetration test. The lines represent the three replicates of the sample.

For the penetration test, an analytical probe p2 was forced down into each gummy bear sample and penetrated it at a defined rate (1 mm/s) to a defined depth (5.0 mm) with a force of 100 g using a load of 5 kg. The maximum force value on the graph is a measure of the hardness of a gummy. A higher peak load indicates a harder gummy bear with lower penetration. The experiment was performed three times, and the results are presented as the mean ± standard deviation. The graphical view is presented in Figure 6.

**Figure 7:**
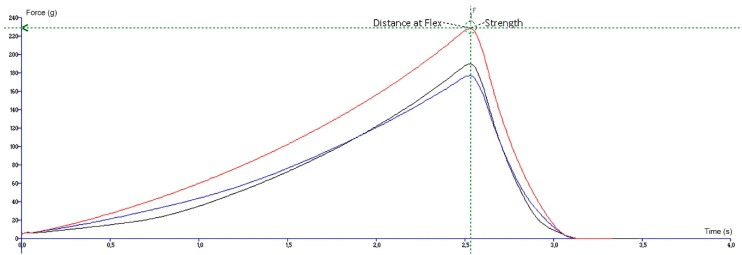
A graphical view of the flexibility test. The lines represent the three replicates of the sample.

A flexibility test using a miniature three-point bend rig (HDP/M3PB) specialized the stress area and stain height parameters so the correct stress and stain could be displayed on the graph axis and the result of the compression and penetration test could be easily rendered. The two adaptable supports for the plate of the rig base were built a suitable distance aside in order to provide the required support for the investigated sample. The distance between the supports was measured and kept constant throughout the experiment. Then, the base plate was fastened to the heavy duty platform. The heavy duty platform was set in the right position if and when the distance between the blade and the two adaptable supports was equal. Further on, the sample was situated in the middle of the supports. The probe was forced down into each gummy bear at 2 mm/s to a depth of 15.0 mm with an automatic trigger force using a load of 5 kg. After the trigger force was reached, the value of force increased and the product started to bend. The gummy bear’s resistance to bending was expressed as the highest point of force. The value of force is associated with the strength of the sample. The measure of a distance at which the force peak is achieved emphasizes the degree of deformation that needs to be applied to the gummy before bending fully commences and specifies flexibility. The experiment was carried out three times, and the obtained results are given as the mean with standard deviation. The graphical view is presented in Figure 7.

### 3.6. The Organoleptic Properties of Gummy Bears Evaluation In Vivo

The organoleptic properties evaluation was conducted according to the protocol of the biomedical study “The chewable dosage form: formulation and quality assessment” (BEC-FF-21) issued by the Lithuanian Bioethics Committee. The trial was carried out in accordance with the Declaration of Helsinki and International Ethical Guidelines for Biomedical Research Involving Human Subjects [51]. The choice of the test methods and search of volunteers were in accordance with ethical guidelines as well. The inclusion criteria were healthy volunteers, speaking and understanding Lithuanian, age above 18 years old, and without a medical history or known allergies to the ingredients present in gummy bear manufacturing. The exclusion criteria were younger than 18 years old, not speaking or understanding Lithuanian, and known allergies to the ingredients used for gummy bear manufacturing. Volunteers signed the Terms of Informed Consent after being informed about the objectives and methods of the research. The participants were informed not to use any mouth hygiene products or eat anything 2 h before and during the study.

Thirteen volunteers participated in the study. The age of the participant varied between 18 and 23 years old. Each participant got four various gummy bear compositions to evaluate according to the following criteria: smell, taste, appearance, color, and firmness. The suggested evaluation was numeric from 1 to 5 (1-hate; 2-dislike; 3-do not mind; 4-like; 5-love). The answers were marked in the questionnaire.

### 3.7. Determination of Calcium Carbonate

The concentration of calcium carbonate in the gummies was determined by complexometric titration. The gummy bear was immersed in a diluted HCl acid and distilled water mixture (ratio 1:3). The flask was heated using a magnetic stirrer (MSH-20A, Witeg Labotechnik GmbH, Wertheim, Germany) until the gummy bear fully dissolved. The neutralization was carried out with NH_4_OH solution following the color change of an indicator (methyl red). Further on, 10–15 mL of the ammonium buffer was added to the neutralized solution. It was titrated with 0.05M EDTA solution and the color change point of eriochrome black was observed. The experiment was repeated with gummy bears stored at room temperature after 7 and 14 days. The values of the calcium carbonate levels were achieved for each sample as the mean ± standard deviation. The experiment was carried out five times.

### 3.8. Determination of Mass Uniformity

The uniformity of mass was determined gravimetrically according to the European Pharmacopoeia 6.0 (01/2008:20905).

Each of the 10 randomly selected gummy bears were carefully placed and weighted using an analytical balance (Mettler Toledo MS 205DU, Switzerland). The gummy bears were then left in an open packaging at room temperature and weighted after 7 and 14 days. The results are presented as mean ± standard deviation. The experiment was carried out three times.

### 3.9. Dissolution Test

The dissolution test was performed at 37 °C in a flask with 100 mL of distilled water using a magnetic stirrer (MSH-20A, Witeg Labotechnik GmbH, Wertheim, Germany). The chronometer was used to follow the dissolution process and determine the dissolution time point. The experiment was repeated with gummy bears stored at room temperature after 7 and 14 days. The results are presented as mean ± standard deviation. The experiment was carried out three times.

### 3.10. Statistical Analysis

The mean and standard deviation of the data were used to present the results of the study. Statistical analysis was carried out using the software package Prism v. 5.04 (GraphPad Software Inc., La Jolla, CA, USA). One-way and two-way ANOVA accompanied by Dunnett’s post-test were applied. The level of significance was 0.05. 

## 4. Conclusions

Based on the obtained texture analysis results, it can be concluded that the optimal composition for the gummy bear’s base contains 4.3 g gelatin and 6.3 g agave syrup. The proposed gummy bear base can be supplemented with various active ingredients, such as calcium carbonate, a solution of vitamin D_3_, and food additives to enhance the acceptance of the product by the consumers. However, the selected color and taste modifier (acai berry extract) did not result in significant improvement of all the organoleptic properties according to the data achieved by the in vivo study. The moisture loss within time was significant as well, so further studies are needed to investigate the other natural sources of food ingredients, which could mask the unpleasant taste of active ingredients if needed and help to avoid water loss (plasticizer effect). 

The results of the current study indicated that experimental texture analysis is a more accurate technique compared to the in vivo evaluation. The proposed analytical methods could be further used for the gummy bear quality evaluation. 

## Figures and Tables

**Table 1:** The influence of added sweeteners on the textural properties of the gummy bears.

Gummy Bears Containing Sweeteners	Texture Properties		
	Firmness (g)	Strength (g)	Hardness (g)
Agave	100.41 ± 2.60	350.39 ± 5.21	704.39 ± 10.31
Xylitol	98.63 ± 3.25	345.36 ± 6.11	700.32 ± 12.54
Sugar	100.12 ± 5.18	351.42 ± 3.98	701.97 ± 9.94

**Table 2:** Statistical data and final equations of responses *.

Response	Min. Value	Max. Value	Mean	SD	Model	*p* Value	Lack of Fit (*p* Value)	R^2^	R^2^ Adjusted	R^2^ Predicted	Final Equation
Firmness (g)	24.71	90.41	50.95	18.48	Quadratic	0.0001	0.2161	0.9958	0.9927	0.9801	=+ 44.15 + 31.25 *A − 2.51 *B − 3.78 *AB + 12.93 *A^2^ + 8.95 *B^2^
Strength (g)	51.64	378.11	177.55	100.34	Quadratic	0.0001	0.7235	0.9866	0.9771	0.9180	=+ 130.75 + 161.97 *A − 27.50 *B − 3.06 *AB + 77.57 *A^2^ − 73.23 *B^2^
Hardness (g)	67.30	726.88	439.29	191.18	Quadratic	0.0172	0.2402	0.9853	0.9748	0.8963	=+ 450.11 + 331.97 *A + 27.79 *B + 35.74 *AB − 75.88 *A^2^ + 42.84 *B^2^

* A-gelatin; B-agave syrup.

**Table 3:** Variables, intervals, and numerical optimization based on surface response design *.

Variables				Responses				
	Low Level (g)	High Level (g)	Predicted Level (g)		Criteria	Importance	Predicted Mean	Observed Mean
Gelatin	1.4	5.6	4.3	Firmness	maximize	++++	61.30	67.15
Agave syrup	0.34	11.7	6.3	Strenght	minimize	++	214.88	230.42
				Hardness	maximize	+++	581.42	564.28

* ++++ maximum importance, + minimum importance.

**Table 4:** Different gummy bear compositions.

Number	Composition	Amount (g)	Number	Composition	Amount (g)
1	Gelatin	4.3	3	Gelatin	4.3
	Water	24.6		Water	24.1
	Agave syrup	6.3		Agave syrup	6.3
	Sorbitol	4.0		Sorbitol	4.0
	Grapefruit seed extract	0.2		Grapefruit seed extract	0.2
	Apple acid	0.6		Apple acid	0.6
				Acai berry extract	0.5
2	Gelatin	4.3	4	Gelatin	4.3
	Water	20.8		Water	20.3
	Agave syrup	6.3		Agave syrup	6.3
	Sorbitol	4.0		Sorbitol	4.0
	Grapefruit seed extract	0.2		Grapefruit seed extract	0.2
	Apple acid	0.6		Apple acid	0.6
	Vit. D3	0.8		Vit. D3	0.8
	Calcium carbonate	3.0		Calcium carbonate	3.0
				Acai berry extract	0.5

**Table 5:** Quality assessment of gummy bears containing calcium carbonate and vitamin D_3_.

Time (Days)	The Concentration of Calcium Carbonate (%)	Mass Uniformity (g)	Dissolution (s)
0	4.8 ± 0.24	3.75 ± 0.18 *	125 ± 20
7	4.8 ± 0.31	2.97 ± 0.13	130 ± 15
14	4.7 ± 0.19	2.11 ± 0.11	133 ± 18

* *p* < 0.05 vs. 7 and 14 days.

**Table 6:** Selected variables and experimental design matrix of gummy bear composition.

Number	Variables		Adjusted Water Content (g)
	Gelatin (g)	Agave Syrup (g)	
1	2.0	10.0	38.0
2	3.5	6.0	36.5
3	5.0	2.0	35.0
4	3.5	6.0	36.5
5	3.5	11.66	36.5
6	5.0	10.0	35.0
7	3.5	6.0	36.5
8	3.5	0.34	36.5
9	1.38	6.0	38.62
10	2.0	2.0	38.0
11	3.5	6.0	36.5
12	5.62	6.0	34.38
13	3.5	6.0	36.5
